# Investigation of ^60^Co Irradiation on the Volatile Organic Compounds from Finger Citron (*Citri Sarcodactylis Fructus*) Using GC–IMS

**DOI:** 10.3390/foods12193543

**Published:** 2023-09-23

**Authors:** Yun Xiang, Chang Lei, Ge Hu, Wei Zhou, Ya Li, Dan Huang

**Affiliations:** 1 State Key Laboratory of Chinese Medicine Powder and Medicine Innovation in Hunan (Incubation), Science and Technology Innovation Center, Hunan University of Chinese Medicine, Changsha 410208, China; xiangyun003997@hnucm.edu.cn (Y.X.); leichang@hnucm.edu.cn (C.L.); huge20220606@163.com (G.H.); fxxx99w@163.com (W.Z.); 2 School of Pharmacy, Hunan University of Chinese Medicine, Changsha 410208, China

**Keywords:** Finger Citron, irradiation sterilization, volatile organic components, chromatography–ion mobility spectrometry

## Abstract

In recent years, as the desire for a healthy lifestyle has become more widespread, consumers are gaining an increasing appreciation for safe, high-quality food. Researchers are constantly seeking new ways to protect foods from insect pests and fungi. This study used GC-IMS to analyze the volatile organic compounds and flavor characteristics of Finger Citron in response to different doses of ^60^Co irradiation. The principal component analysis method was used to explore the overall differences in flavor spectra, and a total of 60 compounds were identified. The fingerprints of volatile organic compounds in the samples showed that the volatile organic compounds with doses of ^60^Co irradiation in about 0 kGy and 5 kGy are similar, while the 10 kGy samples are quite different. The PCA results showed that the similarity between 0 kGy and 5 kGy was slightly higher, and the difference between 10 kGy and other samples was greater. Therefore, it was determined that ^60^Co irradiation with a 10 kGy intensity has a significant influence on the content of volatile oils components, while ^60^Co irradiation with a 5 kGy intensity has little effect. Irradiation technology is demonstrated as a promising method of food sterilization, but the irradiation dose and chemical composition must be taken into consideration.

## 1. Introduction

Finger Citron (*Citri Sarcodactylis Fructus*) is the dried fruit of *Citrus medica* L. var. *sarcodactylis* Swingle. When the fruit turns yellow in autumn, it is collected and is distributed throughout Guangdong, Sichuan, and Zhejiang, China. It is widely used in Chinese food and as a traditional Chinese medicine, which is often used for regulating the liver’s and stomach’s qi flow and relieving pain [1]. Volatile oils (VOs) are substances composed of a variety of compounds, and plant volatile oils can be used to prepare flavors and fragrances, while also possessing strong bacteriostatic activity. Finger Citron volatile oils have obvious inhibitory effects on yeast, *Escherichia coli*, *Bacillus subtilis*, and *Staphylococcus aureus*; they also have an effect on antidepressants and inhibit the reproduction of cancer cells [2,3,4,5]. With regard to the increasingly serious threat of foodborne diseases, plant volatile oils have a very broad development and application prospect as a safe and green high-efficiency food preservative and flavoring agent.

^60^Co has strong bactericidal power, most microorganisms are sensitive to it [6,7,8], and its advantages of convenience and speed are useful during the sterilization of foods and pharmaceutical products. This method destroys microbes in samples and damages DNA in organisms [9]. Under the right conditions, gamma irradiation can effectively destroy mycotoxins and decontaminate plants. To date, the Codex Alimentarius Commission has permitted the decontamination of plant-derived food materials using irradiation in more than 55 countries, including the United States, the European Union, South Korea, and China. In this case, the permitted irradiation dose is lower than 10 kGy. Reviewing the literature, it is evident that there is a great deal of literature on the chemical composition analysis of Finger Citron volatile oils, but very little on using ^60^Co irradiation. Studies have revealed that different irradiation doses can have specific effects on the volatile organic compounds of foods and some Chinese medicinal materials [10,11,12,13].

Compared with GC-MS technology, gas chromatography–ion mobility spectrometry (GC-IMS) has a more efficient separation ability, and with its high selectivity, instrumental simplicity, analytical flexibility, portability, and quasi-real-time monitoring capacity, it can be widely used to analyze the differences in the volatile organic compounds of different products [14,15,16,17,18,19]. In several distinct scientific fields, including air quality control, health assessment, security, and food quality assessment, it has proven to be an effective analytical technique [20,21,22,23,24,25,26]. 

In this study, the volatile flavor substances of Finger Citron volatile oils were analyzed using GC-IMS technology after irradiating at doses of 0, 5, and 10 kGy. Using fingerprint and principal component analyses, the differences and associations of volatile flavor substances under different irradiation doses were explored.

## 2. Materials and Methods

### 2.1. Materials

We collected fresh finger citrons from Jinhua, Zhejiang, China, and Prof. Zhaoming Xie identified them at the Hunan Academy of Traditional Chinese Medicine. A voucher specimen (HNATCM2021-010) was deposited in the herbarium of the Hunan Academy of Traditional Chinese Medicine and stored at 4 °C.

### 2.2. Experimental Methods

#### 2.2.1. ^60^Co Radiation

We then dehydrated the Finger Citron EOs with anhydrous Na_2_SO_4_ and divided them into three equal parts for ^60^Co irradiation (stored in 1.5 mL sealed vials) with a radiation source intensity of 2.96 × 10^16^ Bq using the dynamic stepping irradiation method. The dose rates were 0, 5, and 10 kGy/min. The ^60^Co γ radiation source was located at Hunan Radiological Technology Application Research Center (Changsha, China).

#### 2.2.2. The Extraction Process of Finger Citron Volatile Oils

First, 0.5 kg of Finger Citron was placed in a round-bottomed flask (chopped up before the extraction), which was filled with 3300 mL of water to immerse the Finger Citron. This was extracted via steam distillation and slightly boiled (100 °C) for 5 h. Heating was stopped when the volume of the volatile oil no longer increased. We separated the volatile oils layer, preserved it in a sealed tube, and stored it at 4 °C for analysis.

#### 2.2.3. GC-IMS Analysis

The GC-IMS analysis was performed using a GC-IMS instrument (Flavourspec^®^-G.A.S., Dortmund, Germany). With the carrier gas, samples were introduced into the instrument: first through the gas chromatography column, then into the ion drift tube.

Upon ionization, the molecule migrated to the Faraday disc for secondary separation under the action of an electric field and reverse drift gas.

Samples were loaded into a 20 mL headspace flask, heated at 80 °C for 10 min, and incubated at 500 rpm.

During the headspace injection analysis, the headspace injection volume was 100 μL, and the injection needle temperature was 85 °C.

GC conditions: The column was an MXT-5 column (15 m × 0.53 mm × 1 μm), the column temperature was 60 °C, and N_2_ was used as a carrier gas.

Initially, the carrier gas velocity program was set at 2.0 mL/min, which was maintained for 2 min and then increased linearly to 100.0 mL/min from 2 min to 20 min. After that, it was maintained at 100.0 mL/min from 20 min to 40 min. The flow was stopped after a 40 min runtime.

IMS conditions: N_2_ was the drift gas, and its flow rate was 150 mL/min. Temperature kept at 45 °C.

#### 2.2.4. Statistical Analysis

In order to identify compounds, linear retention indices and mass spectra of GC-IMS data were compared with the NIST 17 database. Our analysis of GC-IMS data (from G.A.S., Dortmund, Germany, version 2.0.0) was performed using Reporter, Gallery Plot, and GC-IMS Library Search. The detected Finger Citron volatile oils were determined by combining the retention index (RI) and drift time (Dt) using NIST Library and IMS database retrieval software from G.A.S (version 2.0.0). This plug-in was used for dynamic PCA, cluster analysis, and a rapid determination of known and unknown samples.

## 3. Results

### 3.1. GC-IMS Profile of Finger Citron at Different Irradiation Doses

**Figure 1:**
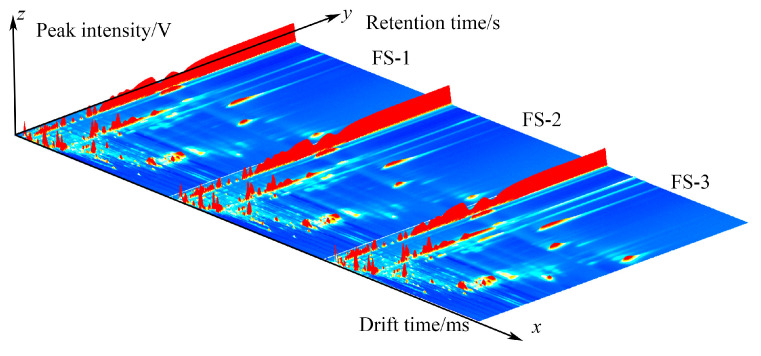
Three-dimensional spectra of volatile organic compounds of three samples: 0 kGy irradiation dose (FS-1), 5 kGy irradiation dose (FS-2), and 10 kGy irradiation dose (FS-3).

A three-dimensional spectrum of volatile organic compounds at ^60^Co doses of 0 kGy, 5 kGy, and 10 kGy is shown in Figure 1, represented by FS-1, FS-2, and FS-3. The *x*, *y*, and *z* axes in the figure represent the drift time, gas chromatography retention time, and peak intensity, respectively. This shows that the peak signal distribution of each group is generally similar, and there is a certain difference in peak signal intensity, indicating that the VOs of Finger Citron volatile oils in each group are generally similar under different irradiation doses, and no new substances are generated. However, there are certain differences in content. By projecting the three-dimensional GC-IMS spectrum, we obtain a two-dimensional GC-IMS plan. Each point represents a volatile oil; the closer the color is to red, the higher the concentration, while the closer it is to white, the lower the concentration. This allows for a more direct evaluation of the volatile species and concentration differences in each sample.

Note: The *x*-axis represents the GC drift time (normalization), the *y*-axis represents the retention time (s), and the *z*-axis represents the intensity of the peak. With blue as the background, the bright spot indicates a substance; the closer its color is to red, the greater the concentration.

**Figure 2:**
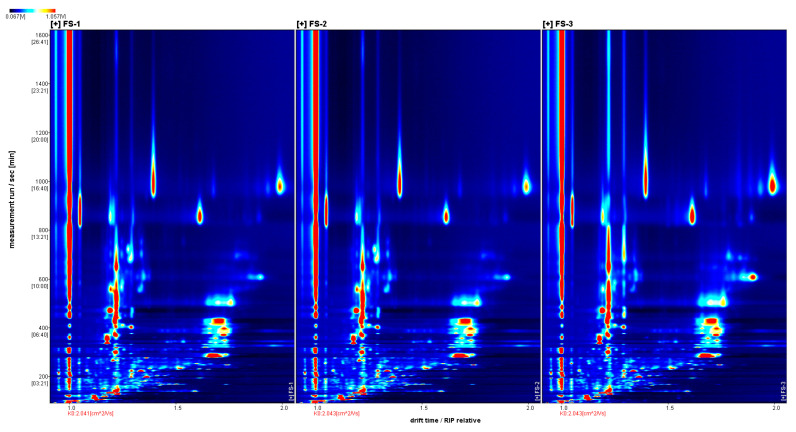
Two-dimensional spectra of volatile substances in three groups of Finger Citron samples. Drift time (normalization) is represented on the *x*-axis; retention time (s) is represented on the *y*-axis.

As shown in Figure 2, the Reporter plug-in was used to generate a two-dimensional top view of Finger Citron volatile oils. It consists of drift time, retention time, and ion signal intensity. This figure has a blue background, and the red vertical line at 1.0 is the RIP (reactive ion peak). Gas chromatography retention time (s) corresponds to the ordinate coordinate and ion drift time (normalization) to the abscissa. Volatile organic compounds are represented by the points on either side of the RIP. The color indicates the concentration of the substance, where white indicates the lowest concentration, red indicates the highest concentration, and a darker color indicates the highest concentration.

**Figure 3:**
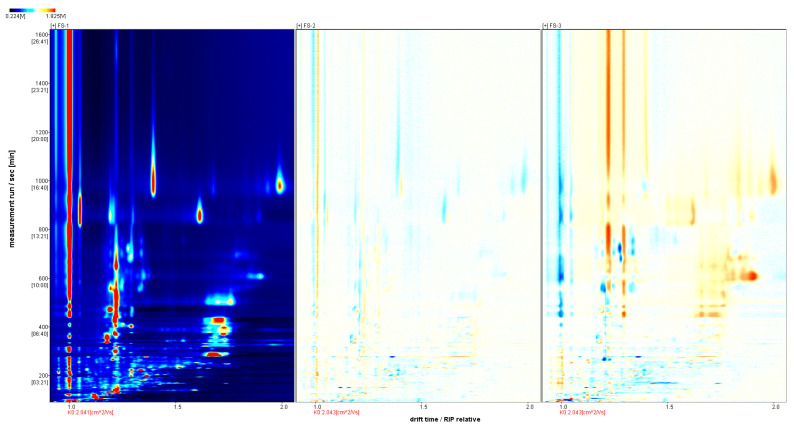
Analysis of the spectral differences between Finger Citron samples from three groups. A comparison of the volatile substance content of different samples was conducted using the FS-1 sample as a reference. The red color indicates a higher concentration of substances in the sample than in the reference sample, whereas the blue color indicates a lower concentration.

Using FS-1 as a reference to establish a difference comparison model, we obtain the results shown in Figure 3. On the basis of Figure 2 spectroscopy, if the volatile organic compounds in the sample are consistent with the volatile organic compounds in the FS-1 sample, the points cancel each other out, and the result is shown in white. If the component is higher than the FS-1 sample, it is shown here more in red and less in blue. FS-2 is mostly red, mixed with a small amount of blue, but the color is relatively light, so the content of FS-2 volatile substances increases and decreases at the same time; however, there is little difference compared to FS-1. There is a significant amount of red and a small amount of blue in FS-3, indicating a large difference in signal strength. Therefore, the irradiation dose can lead to changes in VOs, and the greater the difference in irradiation doses, the more significant the difference in VOs produced.

### 3.2. Qualitative Analysis of Volatile Organic Compounds in Finger Citron (GC × IMS Library Search)

**Figure 4:**
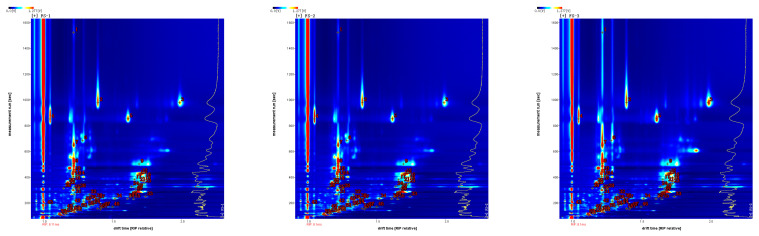
Characteristic peak position plot of volatile organic compounds of Finger Citron at different irradiation doses.

In the GC-IMS 2D pattern, the three samples are reflected in the difference in the content of each volatile substance. On this basis, combined with the NIST and IMS databases built into the software, the volatile organic compounds were qualitatively analyzed, and the ion mobility spectrum of Figure 4 was obtained. Each of these dots represents an organic substance, which was qualitatively searched in the database based on its corresponding two-dimensional data. Drift time is represented by the abscissa, and retention time is represented by the ordinate.

This study identified a total of 60 peak signals, including 10 aldehydes, 10 alcohols, 8 esters, 4 olefins, 4 ketones, 3 acids, and a few pyrazine, pyridine, and furan compounds. The qualitative analysis results of Finger Citron volatile organic compounds are shown in Table 1 and Table 2. 

### 3.3. Principal Component Analysis (PCA) of Volatile Organic Compounds of Finger Citron Samples at 3 Irradiation Doses

**Figure 5:**
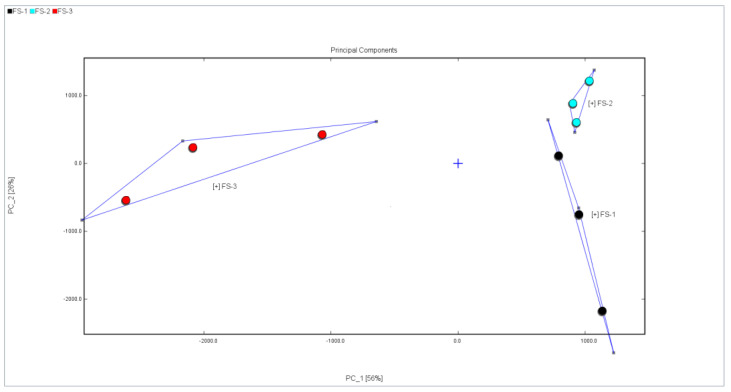
Principal component analysis of volatile organic compounds of Finger Citron in three irradiation doses.

**Figure 6:**
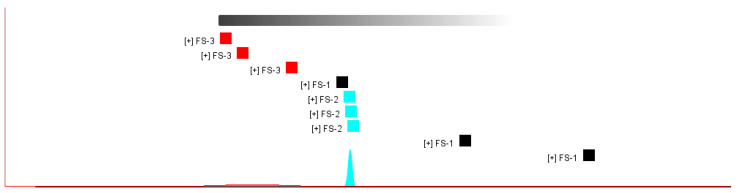
Euclidean distance map of samples’ nearest neighbor. (Compared to distance, similarity is higher the closer the distance.).

The peak volume of 60 volatile organic compounds of three irradiation doses of Finger Citron volatile oils was selected as the characteristic variable for principal component analysis, as demonstrated by Figure 5 and Figure 6. From the figures, it can be concluded that PC1 and PC2 contribute 56% and 26%, respectively (the sum of the contribution rates is 82%). Generally, when the sum of the contribution rates of PC1 and PC2 reaches 60%, the PCA model can fully separate different samples, indicating that principal components 1 and 2 reflect most of the original variable information. The sample distributions with a high correlation will be in the same region; FS-3 is clustered separately from the other two samples, and the PC1 score is negative, indicating that the VOC compositions of FS-1 and FS-2 samples are relatively similar, while the characteristics of FS-3 flavor compounds are significantly different. Thus, three samples can be distinguished via GC-IMS detection of the volatile organic compounds of Finger Citron 0, 5, and 10 kGy irradiated samples.

### 3.4. Fingerprint Analysis of Volatile Organic Compounds of Finger Citron at 3 Irradiation Doses

**Figure 7:**
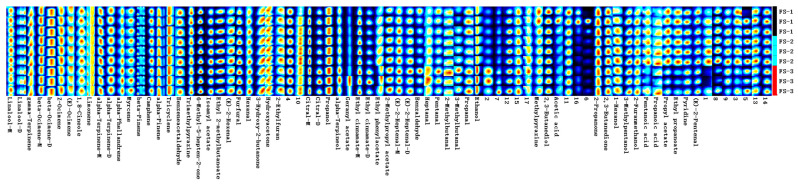
Gallery plot of volatile organic compounds selected via GC-IMS.

The fingerprints of the volatile organic compounds of the three irradiated doses of Finger Citron are shown in Figure 7. GC-IMS analysis revealed that the content of monoterpenes, sesquiterpenes, and alcohols in Finger Citron volatile oils is high, followed by that of alcohols and esters. The fingerprint shows that (E)-2-Pentenal, Pyridine, Ethyl propanoate, Propyl acetate, Acetic acid, Methylpyrazine, and other components gradually decreased from FS-1 to FS-3. Propanoic acid, Pentanoic acid, 2-Furanmethanol, 3-Methylpentanol, 1-Hexanol, and other compounds were most abundant in FS-2 samples. Ethanol, Propanal, 3-Methylbutanal, 2-Methylbutanal, Heptanal, and other compounds had the highest content in FS-3 and the lowest content in FS-1. In this study, the terpenoid components in Finger Citron volatile oils changed, but they remained insignificant, and the active ingredients did not change greatly.

## 4. Discussion

As food and medicine with a high utilization value, Finger Citron volatile oils are an active ingredient [27]. The composition of volatile oils is complex and diverse, often containing dozens or even hundreds of components [28]. The gas chromatography separation technology (GC) and ion migration spectroscopy (IMS) used in this study were able to qualitatively analyze most of the chemical components of Finger Citron volatile oils. The experimental results showed that after irradiation sterilization at three ^60^Co doses of 0 kGy, 5 kGy, and 10 kGy, the volatile organic compounds of FS-1, FS-2, and FS-3 samples were qualitatively analyzed using GC-IMS, and no new chemical substances were produced after ^60^Co irradiation. Analyses of spectrograms and peak volume data revealed that irradiation can affect the content of volatile organic compounds, and different chemical substances will undergo different changes. For example, the content of Geranyl acetate, Ethyl cinnamate, Citral, alpha Terpineol, etc., increased with the increase in irradiation dose; Ethyl phenylacetate, Linalool, Z-Ocimene, and others had the highest content at a radiation dose of 5 kGy ^60^Co, followed by that of 10 kGy, but there was no significant difference between them and that of 0 kGy; Methylpyrazine, 2,3-Butanediol, Propyl acetate, etc., had the highest content at 0 kGy. Therefore, this study concludes that the chemical composition of Finger Citron volatile oils only changes in response to an irradiation dose of 10 kGy ^60^Co, and GC-IMS technology is suitable for a component analysis of volatile oils. As a widely used traditional Chinese medicine with multiple functions, Finger Citron will be subjected to more in-depth research on the specific changes in the content of its active ingredients to determine whether it will lead to changes in pharmacological effects, and the gradient selection of increased irradiation. We will explore this further in future research.

## 5. Conclusions

In this study, GC-IMS was used to detect the volatile organic compounds of Finger Citron volatile oils after sterilization at irradiation doses of 0, 5, and 10 kGy. Based on the built-in NIST gas retention index database and the IMS drift time database, the volatile organic compounds of three volatile oils samples were qualitatively analyzed, and it was determined that the content of terpenes in Finger Citron volatile oils was the highest. By analyzing the GC-IMS fingerprint, PCA, and adjacent Euclidean distance map of the sample, and comparing the content differences of FS-1, FS-2, and FS-3, it can be concluded that irradiation does have a specific effect on the volatile organic compounds of Finger Citron volatile oils, and the larger the irradiation dose gap, the greater the impact on the content of the compound; however, no new compounds are generated. Comparing the fingerprints of the three irradiation doses of Finger Citron volatile oils, it can be observed that a 10kGy ^60^Co irradiation dose has a greater effect on (E)-2-Pentenal, Pyridine, Ethyl propanoate, Propyl acetate, Acetic acid, Methylpyrazine, and other compounds, but less of an impact on terpenes. In summary, this study suggests that ^60^Co irradiation will affect the components of Finger Citron volatile oils, but the impact on its main components is small, and the moderate measurement of ^60^Co irradiation sterilization can be used in the sterilization of Finger Citron medicinal materials, providing a certain reference for the storage and processing of Chinese medicinal materials [9].

GC-IMS has a rapid response time and efficient separation capabilities [29,30,31,32,33]. GC-IMS technology can be used to analyze volatile organic compounds of different products, and it can be used to distinguish isomers effectively, so it is useful for assessing food quality [31,32,33,34].

In today’s world, in which food safety issues are so highly valued, irradiation sterilization technology has undergone over one hundred years of development [35]. It can sterilize without damaging nutritional components, improve hygiene quality, and even improve the flavor of food to a certain extent. It is widely used in the food and drug processing industry [33,34,35,36,37]. However, an improper selection of the irradiation dose can sometimes lead to changes in pharmacological effects and nutritional components [38,39]. Therefore, it is of great significance to study whether this technology is suitable for the sterilization of bergamot and to choose the optimal sterilization dose while maintaining the quality of the food.

## Figures and Tables

**Table 1:** Results of component analysis of Finger Citron volatile oils.

Count	Compound	CAS	Molecular Formula	RI	Rt/s	Dt/ms (RIPrel)	Comment
1	Geranyl acetate	C105873	C_12_H_20_O_2_	1815.8	1521.204	1.22204	-
2	Ethyl cinnamate	C103366	C_11_H_12_O_2_	1438.3	979.259	1.39763	Monomers
3	Ethyl cinnamate	C103366	C_11_H_12_O_2_	1438.3	979.259	1.9898	Dimers
4	Citral	C5392405	C_10_H_16_O	1350.0	852.464	1.04831	Monomers
5	Citral	C5392405	C_10_H_16_O	1351.4	854.509	1.61619	Dimers
6	Ethyl phenylacetate	C101973	C_10_H_12_O_2_	1232.0	683.103	1.28529	-
7	alpha-Terpineol	C98555	C_10_H_18_O	1208.0	648.582	1.22095	-
8	Linalool	C78706	C_10_H_18_O	1107.4	504.171	1.22218	Monomers
9	Linalool	C78706	C_10_H_18_O	1106.2	502.483	1.69512	Dimers
10	gamma-Terpinene	C99854	C_10_H_16_	1066.8	445.944	1.21965	-
11	beta-Ocimene	C13877913	C_10_H_16_	1052.3	425.051	1.21421	Monomers
12	beta-Ocimene	C13877913	C_10_H_16_	1054.1	427.662	1.70437	Dimers
13	Z-Ocimene	C3338554	C_10_H_16_	1038.2	404.81	1.21829	-
14	Benzeneacetaldehyde	C122781	C_8_H_8_O	1042.3	410.686	1.25097	-
15	(E)-Ocimene	C3779611	C_10_H_16_	1045.0	414.604	1.69211	-
16	1,8-Cineole	C470826	C_10_H_18_O	1033.7	398.281	1.73024	-
17	Limonene	C138863	C_10_H_16_	1021.4	380.653	1.6608	-
18	alpha-Terpinene	C99865	C_10_H_16_	1014.6	370.859	1.21693	Monomers
19	alpha-Terpinene	C99865	C_10_H_16_	1015.0	371.512	1.72751	Dimers
20	Trimethylpyrazine	C14667551	C_7_H_10_N_2_	1003.6	355.189	1.17608	-
21	alpha-Phellandrene	C99832	C_10_H_16_	1006.4	359.107	1.68939	-
22	6-Methyl-5-hepten-2-one	C110930	C_8_H_14_O	988.6	338.213	1.18017	-
23	Myrcene	C123353	C_10_H_16_	999.5	349.313	1.7316	-
24	beta-Pinene	C127913	C_10_H_16_	976.2	327.767	1.64037	-
25	Camphene	C79925	C_10_H_16_	946.9	302.956	1.21693	-
26	alpha-Pinene	C80568	C_10_H_16_	932.2	290.551	1.67033	-
27	Tricyclene	C508327	C_10_H_16_	923.7	283.369	1.66761	-
28	(E)-2-Heptenal	C18829555	C_7_H_12_O	955.4	310.138	1.25778	Monomers
29	(E)-2-Heptenal	C18829555	C_7_H_12_O	957.2	311.703	1.67193	Dimers
30	Benzaldehyde	C100527	C_7_H_6_O	961.1	315.009	1.14929	-
31	Pentanoic acid	C109524	C_5_H_10_O_2_	903.0	265.829	1.22569	-
32	Heptanal	C111717	C_7_H_14_O	902.0	265.002	1.33482	-
33	Isoamyl acetate	C123922	C_7_H_14_O_2_	877.2	248.884	1.74347	-
34	(E)-2-Hexenal	C6728263	C_6_H_10_O	847.8	233.593	1.52035	-
35	Ethyl 2-methylbutanoate	C7452791	C_7_H_14_O_2_	843.0	231.113	1.65374	-
36	Furfural	C98011	C_5_H_4_O_2_	827.9	223.26	1.33361	-
37	Methylpyrazine	C109080	C_5_H_6_N_2_	832.7	225.74	1.39545	-
38	2-Furanmethanol	C98000	C_5_H_6_O_2_	849.4	234.419	1.37848	-
39	3-Methylpentanol	C589355	C_6_H_14_O	846.2	232.766	1.6113	-
40	Hexanal	C66251	C_6_H_12_O	805.6	211.688	1.29481	-
41	2,3-Butanediol	C513859	C_4_H_10_O_2_	781.1	199.29	1.36392	-
42	2-Methylpropyl acetate	C110190	C_6_H_12_O_2_	771.3	195.306	1.21788	-
43	Pyridine	C110861	C_5_H_5_N	742.8	183.678	1.01998	-
44	(E)-2-Pentenal	C1576870	C_5_H_8_O	748.2	185.905	1.36201	-
45	3-Hydroxy-2-butanone	C513860	C_4_H_8_O_2_	734.2	180.215	1.33799	-
46	Propyl acetate	C109604	C_5_H_10_O_2_	708.7	169.825	1.50272	-
47	Ethyl propanoate	C105373	C_5_H_10_O_2_	706.9	169.082	1.43637	-
48	Pentanal	C110623	C_5_H_10_O	696.0	164.629	1.42493	-
49	Acetic acid	C64197	C_2_H_4_O_2_	651.9	151.937	1.16181	-
50	Hydroxyacetone	C116096	C_3_H_6_O_2_	608.7	140.528	1.22029	-
51	2,3-Butanedione	C431038	C_4_H_6_O_2_	585.8	134.474	1.18545	-
52	Propanol	C71238	C_3_H_8_O	564.6	128.886	1.22775	-
53	2-Propanone	C67641	C_3_H_6_O	503.8	112.821	1.11577	-
54	Ethanol	C64175	C_2_H_6_O	475.6	105.37	1.12323	-
55	Propanal	C123386	C_3_H_6_O	526.7	118.874	1.15932	-
56	2-Methylbutanal	C96173	C_5_H_10_O	663.4	154.964	1.39573	-
57	3-Methylbutanal	C590863	C_5_H_10_O	651.9	151.937	1.41564	-
58	2-Ethylfuran	C3208160	C_6_H_8_O	720.3	174.522	1.31485	-
59	1-Hexanol	C111273	C_6_H_14_O	875.7	248.098	1.6309	-
60	Propanoic acid	C79094	C_3_H_6_O_2_	682.8	160.086	1.26384	-

Note: RI is the retention index, Rt is the retention time, Dt is the drift time, and [RIPrel] refers to the normalization process.

**Table 2:** Area of Finger Citron volatile oils.

Count	Compound	CAS	Molecular Formula	Comment	[+] FS-1	[+] FS-2	[+] FS-3
1	Geranyl acetate	C105873	C_12_H_20_O_2_	-	2174.20	2809.97	3242.02
2	Ethyl cinnamate	C103366	C_11_H_12_O_2_	Monomers	21,007.74	22,228.10	25,490.10
3	Ethyl cinnamate	C103366	C_11_H_12_O_2_	Dimers	10,415.91	10,665.09	15,509.94
4	Citral	C5392405	C_10_H_16_O	Monomers	17,817.99	19,651.39	20,557.82
5	Citral	C5392405	C_10_H_16_O	Dimers	10,485.74	10,952.11	14,868.86
6	Ethyl phenylacetate	C101973	C_10_H_12_O_2_	-	1801.01	2259.74	1868.36
7	alpha-Terpineol	C98555	C_10_H_18_O	-	6645.98	7106.53	8627.20
8	Linalool	C78706	C_10_H_18_O	Monomers	14,479.82	15,034.59	15,002.15
9	Linalool	C78706	C_10_H_18_O	Dimers	18,387.31	17,681.03	19,107.66
10	gamma-Terpinene	C99854	C_10_H_16_	-	4928.03	5324.99	5441.35
11	beta-Ocimene	C13877913	C_10_H_16_	Monomers	6569.52	6771.70	6558.95
12	beta-Ocimene	C13877913	C_10_H_16_	Dimers	10,060.84	10,008.38	10,162.43
13	Z-Ocimene	C3338554	C_10_H_16_	-	1540.19	1642.53	1585.68
14	Benzeneacetaldehyde	C122781	C_8_H_8_O	-	1117.73	1117.20	1082.82
15	(E)-Ocimene	C3779611	C_10_H_16_	-	5078.11	5200.52	5338.61
16	1,8-Cineole	C470826	C_10_H_18_O	-	1305.46	1319.25	1309.89
17	Limonene	C138863	C_10_H_16_	-	5690.72	5756.64	5768.12
18	alpha-Terpinene	C99865	C_10_H_16_	Monomers	4689.79	4884.76	4749.27
19	alpha-Terpinene	C99865	C_10_H_16_	Dimers	2514.61	2567.02	2700.57
20	Trimethylpyrazine	C14667551	C_7_H_10_N_2_	-	7641.59	7333.87	7059.73
21	alpha-Phellandrene	C99832	C_10_H_16_	-	1296.22	1333.11	1453.98
22	6-Methyl-5-hepten-2-one	C110930	C_8_H_14_O	-	4046.45	4175.84	4391.30
23	Myrcene	C123353	C_10_H_16_	-	1333.17	1307.14	1426.65
24	beta-Pinene	C127913	C_10_H_16_	-	6194.19	6336.64	6305.77
25	Camphene	C79925	C_10_H_16_	-	4119.98	4157.74	4010.99
26	alpha-Pinene	C80568	C_10_H_16_	-	6692.03	6853.91	6727.11
27	Tricyclene	C508327	C_10_H_16_	-	6656.68	6753.87	6696.71
28	(E)-2-Heptenal	C18829555	C_7_H_12_O	Monomers	325.31	345.18	353.34
29	(E)-2-Heptenal	C18829555	C_7_H_12_O	Dimers	181.60	198.59	242.31
30	Benzaldehyde	C100527	C_7_H_6_O	-	450.69	293.64	461.70
31	Pentanoic acid	C109524	C_5_H_10_O_2_	-	659.09	738.71	466.59
32	Heptanal	C111717	C_7_H_14_O	-	130.78	198.43	226.07
33	Isoamyl acetate	C123922	C_7_H_14_O_2_	-	611.47	798.39	808.40
34	(E)-2-Hexenal	C6728263	C_6_H_10_O	-	636.96	874.07	904.80
35	Ethyl 2-methylbutanoate	C7452791	C_7_H_14_O_2_	-	236.78	266.03	264.10
36	Furfural	C98011	C_5_H_4_O_2_	-	2210.18	2478.32	2603.42
37	Methylpyrazine	C109080	C_5_H_6_N_2_	-	312.63	259.44	254.56
38	2-Furanmethanol	C98000	C_5_H_6_O_2_	-	465.39	510.06	502.23
39	3-Methylpentanol	C589355	C_6_H_14_O	-	312.24	356.44	355.38
40	Hexanal	C66251	C_6_H_12_O	-	516.35	566.50	571.58
41	2,3-Butanediol	C513859	C_4_H_10_O_2_	-	3883.92	3213.42	3119.87
42	2-Methylpropyl acetate	C110190	C_6_H_12_O_2_	-	422.88	494.24	543.61
43	Pyridine	C110861	C_5_H_5_N	-	94.87	95.94	84.64
44	(E)-2-Pentenal	C1576870	C_5_H_8_O	-	262.16	251.43	222.98
45	3-Hydroxy-2-butanone	C513860	C_4_H_8_O_2_	-	190.57	196.83	201.22
46	Propyl acetate	C109604	C_5_H_10_O_2_	-	450.26	447.78	253.98
47	Ethyl propanoate	C105373	C_5_H_10_O_2_	-	233.85	237.53	120.04
48	Pentanal	C110623	C_5_H_10_O	-	232.90	186.91	261.11
49	Acetic acid	C64197	C_2_H_4_O_2_	-	382.69	273.02	303.89
50	Hydroxyacetone	C116096	C_3_H_6_O_2_	-	3736.68	3757.55	3624.16
51	2,3-Butanedione	C431038	C_4_H_6_O_2_	-	3617.12	3562.79	3445.12
52	Propanol	C71238	C_3_H_8_O	-	3481.21	3628.86	3843.26
53	2-Propanone	C67641	C_3_H_6_O	-	7010.10	6963.47	7286.70
54	Ethanol	C64175	C_2_H_6_O	-	2742.83	2898.58	2672.49
55	Propanal	C123386	C_3_H_6_O	-	472.26	422.22	485.95
56	2-Methylbutanal	C96173	C_5_H_10_O	-	78.53	81.57	102.75
57	3-Methylbutanal	C590863	C_5_H_10_O	-	53.87	69.16	102.84
58	2-Ethylfuran	C3208160	C_6_H_8_O	-	193.64	200.94	200.05
59	1-Hexanol	C111273	C_6_H_14_O	-	293.47	383.70	377.24
60	Propanoic acid	C79094	C_3_H_6_O_2_	-	146.73	173.43	170.25

## Data Availability

The data used to support the findings of this study can be made available by the corresponding author upon request.
